# Statistical Relationships Between Phonological Form, Emotional Valence and Arousal of Spanish Words

**DOI:** 10.5334/joc.366

**Published:** 2024-05-10

**Authors:** Greig I. de Zubicaray, José A. Hinojosa

**Affiliations:** 1 School of Psychology and Counselling, Faculty of Health, Queensland University of Technology (QUT), Brisbane, Australia; 2 Departamento de Psicología Experimental, Procesos Cognitivos y Logopedia, Universidad Complutense de Madrid, Madrid, Spain; 3 Instituto Pluridisciplinar, Universidad Complutense de Madrid, Madrid, Spain; 4 Centro de Investigación Nebrija en Cognición (CINC), Universidad Nebrija, Madrid, Spain

**Keywords:** Sound symbolism, emotion, valence, arousal, Spanish

## Abstract

A number of studies have provided evidence of limited non-arbitrary associations between the phonological forms and meanings of affective words, a finding referred to as affective sound symbolism. Here, we explored whether the affective connotations of Spanish words might have more extensive statistical relationships with phonological/phonetic features, or *affective form typicality*. After eliminating words with poor affective rating agreement and morphophonological redundancies (e.g., negating prefixes), we found evidence of significant form typicality for emotional valence, emotionality, and arousal in a large sample of monosyllabic and polysyllabic words. These affective form-meaning mappings remained significant even when controlling for a range of lexico-semantic variables. We show that affective variables and their corresponding form typicality measures are able to significantly predict lexical decision performance using a megastudy dataset. Overall, our findings provide new evidence that affective form typicality is a statistical property of the Spanish lexicon.

It is well established that speakers across cultures are able to convey emotional meaning and intent by varying the nonverbal prosodic features of their utterances, such as stress, intonation, pitch, rhythm, and rate (e.g., Frick, 1985; Neves et al., 2021). More recent research has also shown that non-arbitrary relationships between the sounds of words and their meanings convey emotions within and across languages. This *affective sound symbolism* has been shown to manifest cross-linguistically through an over-representation of certain phonemes in words denoting positive or negative emotional valence (the hedonic tone/pleasantness of a word referent; e.g., Adelman et al., 2018; Louwerse & Qu, 2017). For example, the phoneme /i/ is more likely to be associated with positive valence across various languages while nasal (i.e., /n/ or /m/) phonemes are more likely to be associated with negative meanings (e.g., Adelman et al., 2018; Calvillo-Torres et al., 2024; de Zubicaray et al., 2023; Körner & Rummer, 2023; Louwerse & Qu, 2017). The former mapping has been interpreted as evidence for grounding of emotional meaning in bodily or interoceptive experience, following the observation that the muscle used for smiling is also involved in articulating the phoneme /i/ (e.g., Rummer et al., 2014; Sidhu & Pexman, 2018; but see Wagenmakers et al., 2016). A “negative priority” for affective sound symbolism has also been proposed, based on the observation that negative phonemes tend to be uttered more quickly than positive ones, which might reflect an adaptation for alarm signalling (Adelman et al., 2018).

Beyond sound symbolic relationships, there are non-arbitrary relationships between sound and meaning that manifest as statistical regularities more extensively *within* languages, variously referred to as phonological form *systematicity* or *typicality* (see Dingemanse et al., 2015; Haslett & Cai, 2023).^1^ These too have recently been investigated for emotional valence and arousal (the degree to which the activation or intensity of a word’s referent is calming or exciting) at the level of phonetic features (e.g., place and manner of articulation, voicing; Adelman et al., 2018; Benczes & Kovács, 2022; Calvillo-Torres et al., 2024; de Zubicaray et al., 2023; Kambara & Umemura, 2021; Louwerse & Qu, 2017). In English, positively valenced words tend to have more bilabial and velar sounds in their initial phonemes and more labiodental final phonemes, while negatively valenced words are more likely to comprise more stops and fricatives and have a stressed syllable in addition to more nasal sounds in their initial phoneme (e.g., de Zubicaray et al., 2023; Louwerse & Qu, 2017). In Spanish, approximants tend to occur in positive and low arousing words, while fricatives are overrepresented in negative words and in those denoting high arousal, with the latter forms also tending to have more plosives (Calvillo-Torres et al., 2024). In German, words expressing high arousing concepts tend to comprise short vowels, voiceless consonants, and hissing sibilants (Aryani et al., 2018; Schmidtke & Conrad, 2018; Ullrich et al., 2016). In Hungarian, a non-Indo-European language, positive valence instead tends to be associated with more fricatives, palatals and sibilant sounds and negative valence with plosives (Benczes & Kovács, 2022). However, it should be acknowledged these statistical regularities explain a relatively small proportion of variance in affective ratings (i.e., a few percent; e.g., Benczes & Kovács, 2022; de Zubicaray et al., 2023).

Researchers interested in investigating systematic affect-form mappings face several issues that can compromise the validity of their findings, such as the sizes of available affective ratings norms, evidence of substantial disagreement in affective ratings for certain words, and morphophonological redundancies, as well as the generalization of phonological form typicality effects within and across languages. The majority of studies of emotional sound symbolism have entailed relatively small samples of words ranging from several hundred to several thousand. Such samples are unlikely to accurately reflect the full extent of sound-meaning mappings within a language. In an attempt to circumvent this issue, some studies have simply extrapolated Warriner et al.’s (2013) affective ratings for English words to translated forms in other languages (e.g., Louwerse & Qu, 2017). Yet, such an approach neglects the evidence of considerable variability in the affective meaning of translated words across cultures, especially across those that are more geographically or linguistically distant from each other (e.g., Jackson et al., 2019).

To identify valid form-meaning mappings at the lexicon level, it is essential that affective connotations be agreed upon by the majority of language users. Even with large samples of words (~ 14,000) such as those available for English (Warriner et al., 2013) and Spanish (Stadthagen-Gonzalez et al., 2017), the use of averaged subjective ratings will introduce substantial noise in analyses if participants vary considerably in their responses to a given word, as indicated by a large rating standard deviation (see Pollock, 2018). This is because words that evoke disparate responses across participants invariably result in average ratings within the middle of the scale, which constitute the majority of words. This issue affects almost all studies of affective sound symbolism. For example, de Zubicaray et al. (2023) showed that approximately two thirds of the words in the Warriner et al. (2013) English norms had poor inter-rater agreement for valence. They were also unable to investigate form-meaning mappings for arousal as only 86 words showed reasonable rating agreement, raising questions about the validity of prior research in English that used this measure. Deriving word meaning representations from contextual co-occurrence vectors instead of subjective ratings (e.g., Monaghan et al., 2014; Recchia & Louwerse, 2015) is also problematic, as these measures are relatively poor at estimating the extremes of human judgements and potentially introduce artefactual values (Hollis, Westbury, & Lefsrud, 2017; Mandera, Keuleers, & Brysbaert, 2015). Even though extreme ratings are the most useful for distinguishing valence and arousal, focussing exclusively on these subsets also increases the risk of revealing relationships that are not representative of the full lexicon (e.g., Keuleers & Balota, 2015; Liben-Nowell et al., 2019). Finally, Adelman et al. (2018) noted that morphophonological redundancy in negating prefixes in English words such as “in-” and “un-” as in *inedible* or *unhappy* are likely to skew findings (see also de Zubicaray et al., 2023). Across languages, negation is almost invariably prefixal (e.g., Cartoni & Lefer, 2011).

In the present study, we aimed to investigate phonological form typicality for affective connotations in Spanish using the rating norms for 14,031 words provided by Stadthagen-Gonzalez et al. (2017), adopting a similar approach to that used by de Zubicaray et al. (2023) for English words. While English and Spanish share many sounds and so might be expected to yield similar results, there are also key differences (Fabiano-Smith & Goldstein, 2010). For example, English has more than 10 phonetic vowels while Spanish has only five. Phonetic consonants also differ across the two languages. The English consonants */v/, /z/, /ò/* and */*ɹ*/* do not occur in Spanish, while the trilled consonant /*r*/ does not occur in English (Carlo, Wilson, & Villanueva-Reyes, 2020). Spanish is also more phonotactically constrained than English, having fewer onset consonant clusters as well as word endings that do not have coda clusters. Consequently, monosyllabic words are much less frequent in Spanish than in English (Carlo et al., 2020). This means that approaches devised to study phonological similarity in solely monosyllabic English words are not applicable to Spanish (e.g., Monaghan et al., 2014). In addition, Spanish has more regular spelling-to-sound mappings than English such that its orthography is usually characterized as shallow or transparent (Rodríguez-Ferreiro & Davies, 2019).

Two previous studies reported evidence of affective sound symbolism in Spanish (Adelman et al., 2018; Calvillo-Torres et al., 2024) using the Stadthagen-Gonzalez et al. (2017) norms. Calvillo-Torres et al. (2024) examined relationships between affective dimensions and discrete phonemes (*n* = 31), grouping phonemes with significant form-meaning mappings according to their phonetic features. Adelman et al.’s (2018) study examined relationships with phonemes and phonetic features. However, neither controlled for affective rating disagreement or redundant affixes or investigated positioning of syllabic stress or variation according to grammatical class. For example, various authors have noted that adjectives directly reference immediate feelings and emotional states (e.g., Béligon, 2020; Galati et al., 2008; Pérez-Sánchez et al., 2021). In English, adjectives are reported to comprise the most typical forms for positive valence (de Zubicaray et al., 2023). In addition, neither investigated extremity of valence or *emotionality* (i.e., the absolute distance from the midpoint of the valence rating scale, regardless of polarity, corresponding to the quadratic term; e.g., Adelman & Estes, 2013). For example, de Zubicaray et al. (2023) reported that more emotionally intense words in English tend to be associated with voiced sounds, while nasal sounds were over-represented in the first phonemes of more neutral words. Their combined findings therefore suggested a qualified relationship in which nasal initial phonemes signalled negative valence in English only when additionally stressed.

A second aim of our study was to investigate whether affective form typicality might influence lexical processing in Spanish by capitalising on recent behavioural megastudy data acquired with the visual lexical decision task (LDT; Haro et al., 2024). To our knowledge, only two studies have investigated the influence of affective variables on Spanish word processing with the LDT using megastudy data. Rodríguez-Ferreiro and Davies (2019) reported a graded effect of valence on response times, with positive words responded to more quickly than neutral and negative words. However, they did not observe an effect of arousal. Haro et al. (2024) recently replicated the graded effect of valence on RTs, and observed an effect of arousal, as well as an interaction between arousal and valence. However, neither study controlled for rating disagreement nor investigated emotionality (e.g., Pollock, 2018). In English, affective variables and their corresponding form typicality values have been shown to be relatively weak and opposing predictors of visual LDT RTs, with more typical forms slowing responses (see de Zubicaray et al., 2023). However, it is possible that stronger form typicality effects in the LDT might be observed in Spanish as it entails phonological recoding more so than English due to its shallow orthography (Álvareza, Taft, & Hernández-Cabrera, 2017).

## Study 1: Investigating form-affect mappings of Spanish words

**Figure 1:**
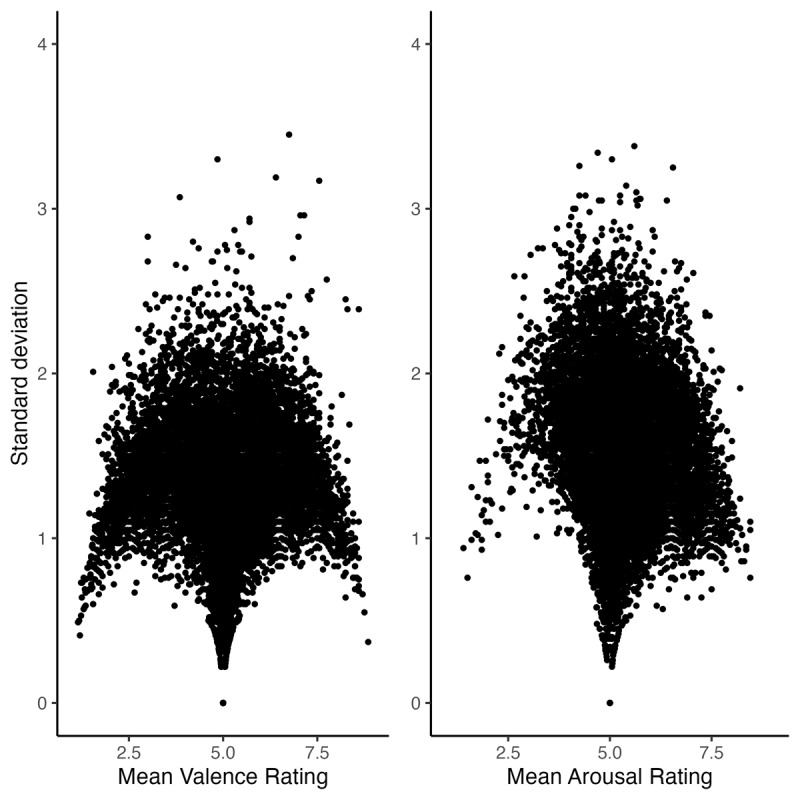
Valence and arousal ratings agreement in the Stadthagen-Gonzalez et al. norms (*N* = 14,028).

To investigate systematic form-affect mappings in Spanish, we used Stadthagen-Gonzalez et al.’s (2017) norms for 14,028 monosyllabic and polysyllabic words. Valence and arousal were rated on nine-point scales from *infeliz* (unhappy) to *feliz* (happy), and *tranquilo* (quiet) to *exitado* (excited), respectively. Stadthagen-Gonzalez et al. (2017) reported their average standard deviations for these ratings were 1.27 and 1.50, respectively, which compare quite favourably with the larger standard deviations reported for Warriner et al.’s (2013) English word norms (1.68 and 2.30, respectively). The mean valence and arousal values and corresponding standard deviation of every Spanish word rated in Stadthagen-Gonzalez et al.’s (2017) norms are plotted in Figure 1. As the plots show, participants disagreed about the valence and arousal ratings for a sizeable proportion of words. As Pollock (2018) noted, a standard deviation above 1.5 “means that some people report a very strong negative response to that word, whereas some people report little or no emotional response at all. So if a researcher is interested in comparing responses to neutral words with responses to emotionally valenced words, they should definitely avoid words with high standard deviations for emotional valence, because they will add a significant amount of noise to the experimental design” (p. 1212). We therefore used a cutoff of 1.5 standard deviations to identify words with reasonable rating agreement for both valence and arousal (see de Zubicaray et al., 2023). In addition, we derived measures of form typicality for each affective rating and investigated whether they varied according to Part-of-Speech (Duchon et al., 2013).

## Methods

### Materials

We used the EsPal database (Duchon et al., 2013; token subtitle) to restrict the list of words with reasonable (i.e., SD < 1.5) rating agreement for both valence and arousal in Stadthagen-Gonzalez et al.’s (2017) norms to their maximum lemmas. Next, we used EsPal’s Part-of-Speech category to exclude any words corresponding to numbers, proper names, adpositions, dates, determiners, interjections or pronouns. This process resulted in a list of 3915 words with reasonable rating agreement for both valence and arousal. Next, removal of prefixed words from the dataset resulted in a final list of 3669 words, comprising 2369 nouns, 560 adjectives, 693 verbs, and 47 adverbs.^2^

Phonemic transcriptions (*es_phon_structure*) and lexical stress position (*es_syll_accent*) assignments were also taken from EsPal. We coded 80 form variables for each word: numbers of letters, phonemes and syllables, initial and final phonemes (a number was assigned to each of the 31 Espal phonemes), the number of typical phonetic features (i.e., place and height for vowels; place and manner of articulation for consonants; voicing), phonetic features occurring in initial and final positions, and the position of the syllable with primary lexical stress: initial, final, and medial. We included orthographic length (number of letters) as speech signal durations were unavailable. To calculate Emotionality, we subtracted the midpoint of the scale (5) from the Stadthagen-Gonzalez et al.’s (2017) valence rating of each word and eliminated its polarity.

### Design and Analysis

To investigate systematic form-affect relationships in Spanish words, we adopted the same three-step approach to that applied by de Zubicaray et al. (2023) in English using R (version 4.3.1; R Core Team, 2023): We first excluded form variables with zero variance and linear dependencies (*caret* package – *findLinearCombos*; Kuhn, 2022), then determined the best subset of form variables for predicting each of the valence, emotionality, and arousal ratings (*leaps* package; Lumley, 2022). Finally, we used a 10-fold cross-validation procedure (repeated 200 times with different randomised folds; *caret* package) to determine the best-fitting model in terms of predictive accuracy. We selected the model that minimised root mean square error (RMSE) to avoid overfitting (see de Rooij & Weeda, 2020; Yarkoni & Westfall, 2017). To ensure valid coefficient estimates for form variables demonstrating skewness, outliers, multicollinearity and/or heteroscedasticity, we entered the best fit model into a linear regression with robust standard errors (Wilcox, 2019).

*Transparency and openness:* We provide all our data and analysis scripts for this and the subsequent studies at: https://osf.io/mxhnq/.

## Results and Discussion

We calculated measures of form typicality for each of the three affective content ratings following the approach described in de Zubicaray et al. (2023), i.e., we extracted the predicted value of the dependent variable for each word according to the robust regression model (i.e., the fitted values from the matrix of predicted means). The values for all words were then Z-transformed. Hence, positive typicality values indicate word forms aligned with positive valence or arousal and negative values indicate forms with negative valence or arousal. For emotionality ratings, positive typicality values indicate forms with high emotional load regardless of polarity, while negative values indicate more neutral forms. We conducted analyses of variance (ANOVA) to determine whether form typicality for each affect rating varied according to Part-of-Speech. Bartlett’s test showed the valence form typicality data violated the assumption of homogeneity of variance, χ^2^(3) = 27.889, *p* < .001, as did emotionality, χ^2^(3) = 53.723, *p* < .001, and arousal, χ^2^(3) = 59.359, *p* < .001. We therefore conducted Welch’s ANOVAs, followed by Games-Howell post hoc tests (package *rstatix;* Kassambara, 2021) and plotted distributions and probability densities in violin plots (package *ggstatsplot;* Patil, 2021).

## Results and Discussion

Form variables were able to predict a significant proportion of variance in all three ratings of affective content. Tables 1, 2 and 3 provide summaries of the best fit regression models for predicting valence (adjusted *R^2^* of 0.030), emotionality (adjusted *R^2^* of 0.041) and arousal (adjusted *R^2^* of 0.058). We were able to replicate the finding of first and final phoneme positions significantly predicting valence in Spanish (Adelman et al., 2018), as well as Calvillo-Torres et al.’s (2024) findings of nasals in the first phoneme position and fricatives being over-represented in negative words.

**Table 1:** Best fit model for predicting valence with form variables according to 10-fold cross validation repeated 200 times (*n* = 3669).


MODEL	*ESTIMATE*	*STD. ERROR*	*t*

(Intercept)	4.369	0.178	24.489***

Length	–0.226	0.059	–3.847***

Number phonemes	0.169	0.060	2.801**

Number labiodental	–0.139	0.084	–1.664

Number alveolar	0.065	0.029	2.283*

Number fricative	–0.064	0.032	–1.984*

Number affricate	0.243	0.128	1.907

Number lateral	0.089	0.041	2.179*

Number mid	0.067	0.025	2.726**

Number unrounded	0.071	0.028	2.521*

Initial Phoneme	0.006	0.002	2.555*

Final Phoneme	0.026	0.008	3.325***

First Phoneme bilabial	0.122	0.050	2.448*

First Phoneme labiovelar	–2.076	464.000	–0.004

First Phoneme velar	0.145	0.053	2.730**

First Phoneme nasal	–0.204	0.081	–2.515*

Final Phoneme alveolar	–0.503	0.116	–4.335***

Final Phoneme fricative	–0.292	0.156	–1.865

Final Phoneme lateral	0.359	0.092	3.881***

Final Phoneme unrounded	0.528	0.143	3.705***

Final Stress Position	0.497	0.106	4.704***


* p < .05; ** p < .01; *** p < .001.

**Table 2:** Best fit model for predicting emotionality with form variables according to 10-fold cross validation repeated 200 times (*n* = 3669).


MODEL	*ESTIMATE*	*STD. ERROR*	*t*

(Intercept)	0.820	0.054	15.260***

Length	0.098	0.033	2.959**

Number syllables	–0.138	0.045	–3.060**

Number bilabial	–0.109	0.039	–2.786**

Number dental	–0.139	0.041	–3.425***

Number alveolar	–0.149	0.036	–4.191***

Number palatal	–0.212	0.059	–3.580***

Number labiovelar	–0.303	0.077	–3.960***

Number velar	–0.200	0.041	–4.843***

Number nasal	0.065	0.020	3.178**

Number fricative	0.067	0.025	2.743**

Number approximant	0.114	0.028	4.135***

Number voiceless	0.071	0.025	2.877**

Number unrounded	0.026	0.018	1.441

First Phoneme alveolar	–0.074	0.036	–2.036*

First Phoneme labiovelar	1.746	10.667	0.164

First Phoneme voiceless	–0.114	0.031	–3.658***

Final Phoneme dental	0.264	0.103	2.572*

Final Phoneme alveolar	0.791	0.220	3.596***

Final Phoneme nasal	–0.809	0.306	–2.642**

Final Phoneme fricative	–0.336	0.149	–2.253*

Final Phoneme lateral	–0.953	0.224	–4.250***

Final Phoneme trill	–0.600	0.222	–2.700**


* p < .05; ** p < .01; *** p < .001.

**Table 3:** Best fit model for predicting arousal with form variables according to 10-fold cross validation repeated 200 times (*n* = 3669).


MODEL	*ESTIMATE*	*STD. ERROR*	*t*

(Intercept)	5.281	0.112	47.019***

Length	0.070	0.012	6.051***

Number labiodental	0.130	0.059	2.197*

Number alveolar	–0.042	0.022	–1.931

Number velar	–0.057	0.024	–2.312*

Number fricative	0.057	0.024	2.410*

Number affricate	–0.205	0.106	–1.940

Number lateral	–0.150	0.031	–4.774***

Number mid	–0.041	0.019	–2.198*

Number unrounded	–0.043	0.020	–2.115*

First Phoneme dental	0.102	0.049	2.070*

First Phoneme affricate	0.519	0.216	2.399*

First Phoneme voiceless	–0.076	0.034	–2.235*

First Phoneme unrounded	0.183	0.041	4.414***

Final Phoneme alveolar	0.165	0.089	1.850

Final Phoneme lateral	–0.334	0.062	–5.353***

Final Phoneme open	–0.318	0.108	–2.929**

Final Phoneme mid	–0.262	0.109	–2.392*

Final Stress Position	–0.198	0.100	–1.987*


* p < .05; ** p < .01; *** p < .001.

**Figure 2:**
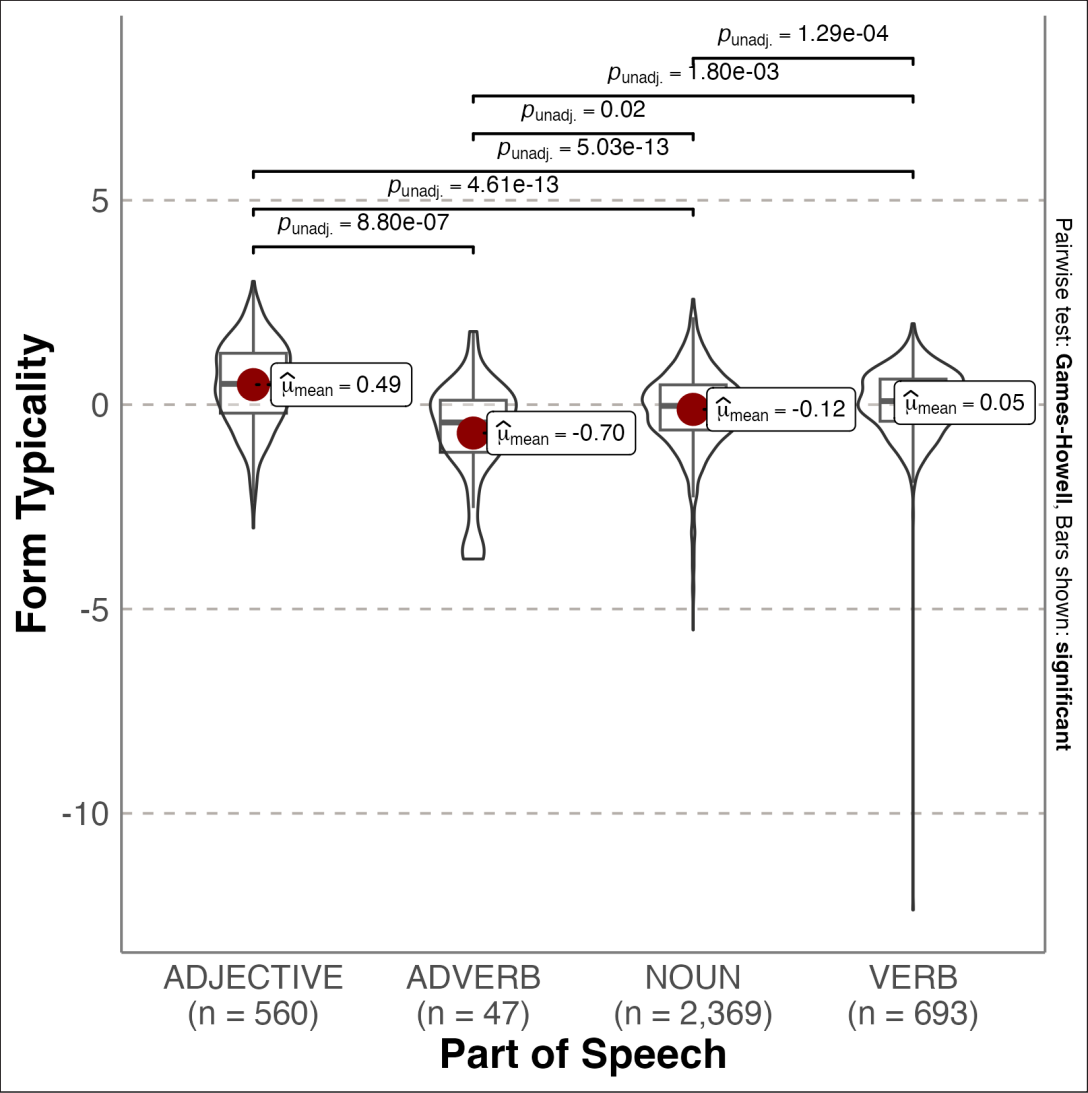
Violin plot showing probability densities of valence form typicality values as a function of Part of Speech. The red dot indicates the mean.

**Figure 3:**
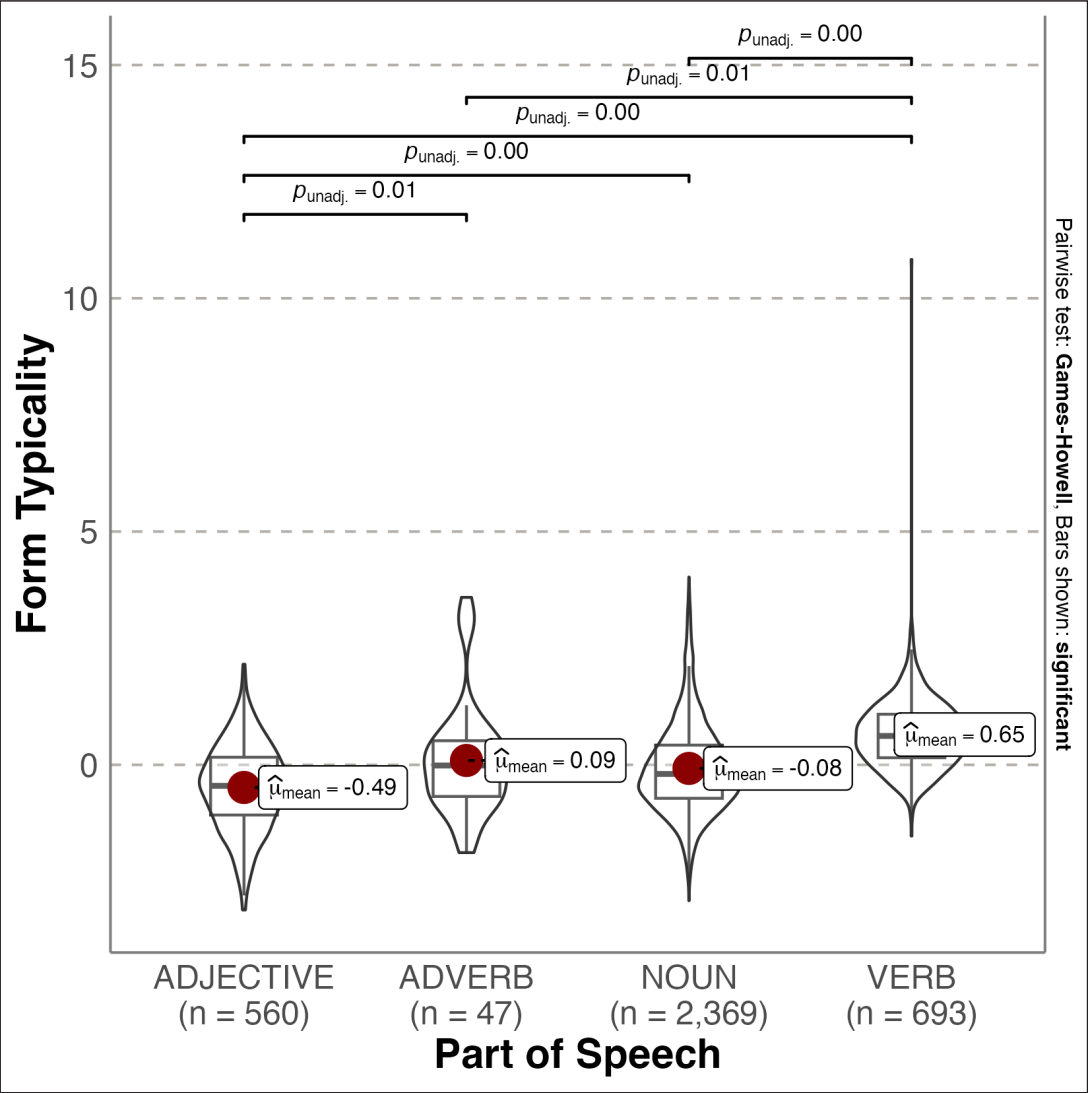
Violin plot showing probability densities of emotionality form typicality values as a function of Part of Speech. The red dot indicates the mean.

**Figure 4:**
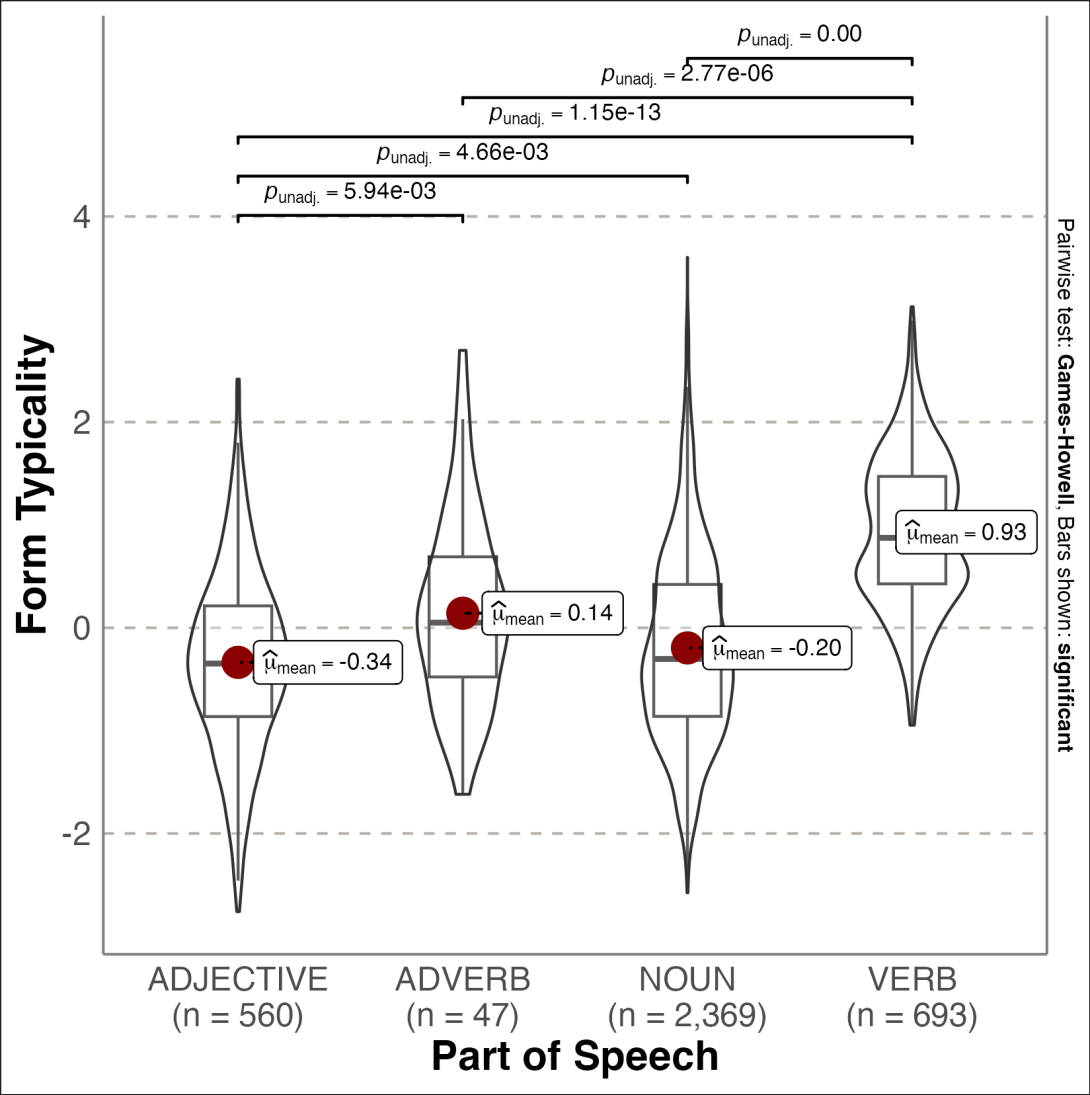
Violin plot showing probability densities of arousal form typicality values as a function of Part of Speech. The red dot indicates the mean.

Figures 2, 3 and 4 show how form typicality for the three types of affective content varies as a function of Part of Speech. Form typicality for valence varied significantly according to Part of Speech, Welch’s *F*(3, 211.24) = 59.265, *p* < .001, ω^2^ = 0.051, with adjectives comprising the most typical forms for positive valence. Post hoc Games-Howell tests revealed adjectives had significantly more typical forms for positive valence than adverbs (Mean_diff_ = 1.184, p < .001), nouns (Mean_diff_ = 0.605, p < .001) and verbs (Mean_diff_ = 0.440, p < .001). Adverbs had significantly more typical forms for positive valence than both nouns (Mean_diff_ = 0.579, p = .02) and verbs (Mean_diff_ = 0.744, p = .002), and verbs significantly more than nouns (Mean_diff_ = 0.165, p < .001).

A different pattern emerged for emotionality, Welch’s *F*(3, 212.28) = 209.11, *p* < .001, ω^2^ = 0.119, with verbs being the most form typical for high emotionality. Adjectives were significantly less typical forms for high emotionality than adverbs (Mean_diff_ = –0.579, p = 0.011), nouns (Mean_diff_ = –0.408, p < .001) and verbs (Mean_diff_ = –1.13, p < .001). Adverbs did not significantly differ from nouns (Mean_diff_ = –0.170, p = .767) but adverbs were significantly less typical forms for high emotionality than verbs (Mean_diff_ = –0.556, p = .015). Nouns were significantly less typical than verbs (Mean_diff_ = –0.726, p < .001).

Arousal form typicality also varied significantly as a function of Part of Speech, Welch’s *F*(3, 213.945) = 420.13, *p* < .001, ω^2^ = 0.205, with verbs’ forms being most typical for high arousal. Adjectives were significantly less typical forms for high arousal than adverbs (Mean_diff_ = –0.478, p = 0.006), nouns (Mean_diff_ = –0.141, p = .005) and verbs (Mean_diff_ = –1.266, p < .001). Adverbs did not significantly differ from nouns (Mean_diff_ = 0.337, p = .073) but were significantly less typical forms for high arousal than verbs (Mean_diff_ = –0.789, p < .001). Nouns were significantly less typical forms for high arousal than verbs (Mean_diff_ = –0.726, p < .001). Overall, the pattern for arousal form typicality closely resembled that for emotionality. Table 4 shows the top 10 most and least typical word forms for valence, emotionality and arousal.

**Table 4:** Words with the 10 most and least form typical values for each of the three affective ratings.


VALENCE				EMOTIONALITY				AROUSAL			
		
MOST TYPICAL		LEAST TYPICAL		MOST TYPICAL		LEAST TYPICAL		MOST TYPICAL		LEAST TYPICAL	
					
WORD	VALUE	WORD	VALUE	WORD	VALUE	WORD	VALUE	WORD	VALUE	WORD	VALUE

general	3.02	huir	–12.36	huir	10.83	contractual	–3.11	apendicitis	3.60	local	–2.76

colateral	2.78	faringitis	–5.51	hepatitis	4.02	cruel	–3.06	especificar	3.12	colateral	–2.61

craneal	2.59	hepatitis	–4.81	oficialidad	3.79	colateral	–3.04	hepatitis	3.11	laurel	–2.58

panel	2.58	tifus	–4.68	enfermedad	3.69	corporal	–2.94	intensificar	2.99	colonial	–2.54

penal	2.58	escurreplatos	–4.56	tifus	3.65	coronel	–2.91	infanticidio	2.97	lateral	–2.46

peral	2.58	conjuntivitis	–4.53	apendicitis	3.61	troncal	–2.80	escurreplatos	2.95	literal	–2.46

coronel	2.56	lavavajillas	–4.52	entonces	3.59	comarcal	–2.71	administrador	2.91	canal	–2.44

poligonal	2.54	abrebotellas	–4.43	abrebotellas	3.51	rural	–2.65	infundir	2.87	poligonal	–2.42

elemental	2.53	apendicitis	–4.39	mantis	3.48	craneal	–2.62	dermatitis	2.87	craneal	–2.38

unilateral	2.52	meningitis	–4.37	amigdalitis	3.36	sexual	–2.58	faringitis	2.85	panel	–2.37


## Study 2: Relationships between form typicality for affective content and lexico-semantic variables

Past work in Spanish has tended to show that lexical frequency is positively correlated with valence (e.g., Hinojosa et al., 2016a, 2023; Pérez-Sánchez et al., 2021; Stadthagen-Gonzalez et al., 2017) although results for arousal have been mixed with some studies reporting negative correlations (e.g., Hinojosa et al., 2016a, 2023; Stadthagen-Gonzalez et al., 2017) and others positive ones (e.g., Guasch et al., 2016; Pérez-Sánchez et al., 2021). In addition, age-of-acquisition (AoA) is positively correlated with arousal ratings but negatively correlated with valence (e.g., Hinojosa et al., 2016b, 2023; Pérez-Sánchez et al., 2021; Stadthagen-Gonzalez et al., 2017; but see Pérez-Sánchez et al., 2021 who failed to observe a significant correlation with arousal). Significant negative correlations have also been reported between concreteness/imageability and valence (e.g., Hinojosa et al., 2023; Pérez-Sánchez et al., 2021; Stadthagen-Gonzalez et al., 2017) and positive correlations with familiarity (e.g., Hinojosa et al., 2023; Stadthagen-Gonzalez et al., 2017). Conversely, arousal has been reported to be negatively correlated with both familiarity and concreteness/imageability (e.g., Guasch et al., 2016; Stadthagen-Gonzalez et al., 2017; but see Pérez-Sánchez et al., 2021 who failed to observe a significant correlation between concreteness and arousal). Emotionality (or “emotional load”) has also been reported to be negatively correlated with concreteness/imageability and familiarity (Guasch et al., 2016) or positively correlated with concreteness (Pérez-Sánchez et al., 2021). We therefore investigated relationships between our measures of affective form typicality from Study 1 and these lexico-semantic variables. We also investigated whether our form typicality measures are still able to predict the Stadthagen-Gonzalez et al., (2017) valence, emotionality and arousal ratings after controlling for the sublexical and lexical variables (e.g., Adelman et al., 2018).

## Methods

### Materials

For each of the 3669 unaffixed words from Study 1, we derived the following lexico-semantic variables in addition to the affective ratings from Stadthagen-Gonzalez et al., (2017): Number of letters,^3^ Orthographic Levenshtein Distance (OLD), mean bigram frequency, number of phonological neighbours, subtitle Zip frequency and Part of Speech were sourced from EsPal (Duchon et al., 2013). We also included a measure of prevalence derived from Castillian Spanish native speakers from Spalex (Aguasvivas et al., 2018). Familiarity, concreteness and Age-of-Acquisition (AoA) were sourced from Haro et al. (2024), which comprised novel ratings from their own study and from various databases for 7500 words (Alonso et al., 2015; Duchon et al., 2013; Ferré et al., 2012; Guasch et al., 2016; Hinojosa et al., 2016a, b; Huete-Pérez et al., 2019). This resulted in a final set of 1862 words (1292 nouns, 181 adjectives, 25 adverbs and 364 verbs) with values for all variables across databases. Descriptive statistics for these variables are summarised in Table 5.

**Table 5:** Descriptive statistics for the variables in Study 2 (*n* = 1862).


VARIABLE	MEAN	SD

Length	7.1	1.90

OLD	1.9	0.60

Mean bigram frequency	5732	3607.00

Phonological Neighbours	9.3	11.00

Subtitle Zipf frequency	3.7	0.77

Age of Acquisition	7.3	2.00

Prevalence	2.3	0.28

Concreteness	4.6	1.10

Familiarity	5.1	1.00

Valence	5	1.30

Emotionality	0.96	0.86

Arousal	5.4	1.00

Form Typicality (Valence)	–0.021	0.98

Form Typicality (Emotionality)	–0.00	1.00

Form Typicality (Arousal)	–0.08	0.99


OLD: Orthographic Levenshtein Distance.

### Design and analysis

We first calculated Spearman correlations between the respective affective ratings, their corresponding form typicality values and the lexico-semantic variables. Next, we performed separate hierarchical linear regressions with robust standard errors (Wilcox, 2016). For each rating as dependent variable, we entered the lexico-semantic variables as control predictors in Step 1. Part of Speech was the only categorical predictor with nouns chosen as reference category as they comprised most words. In Step 2, we added the other two affective ratings (e.g., if valence was dependent variable, we entered emotionality and arousal as predictors). Finally, in Step 3 we added the form typicality measures. All predictor variables were mean-centred. We used the package *lmtest* (Zeileis & Hothorn, 2002) to test each model’s significance.

## Results and discussion

**Figure 5:**
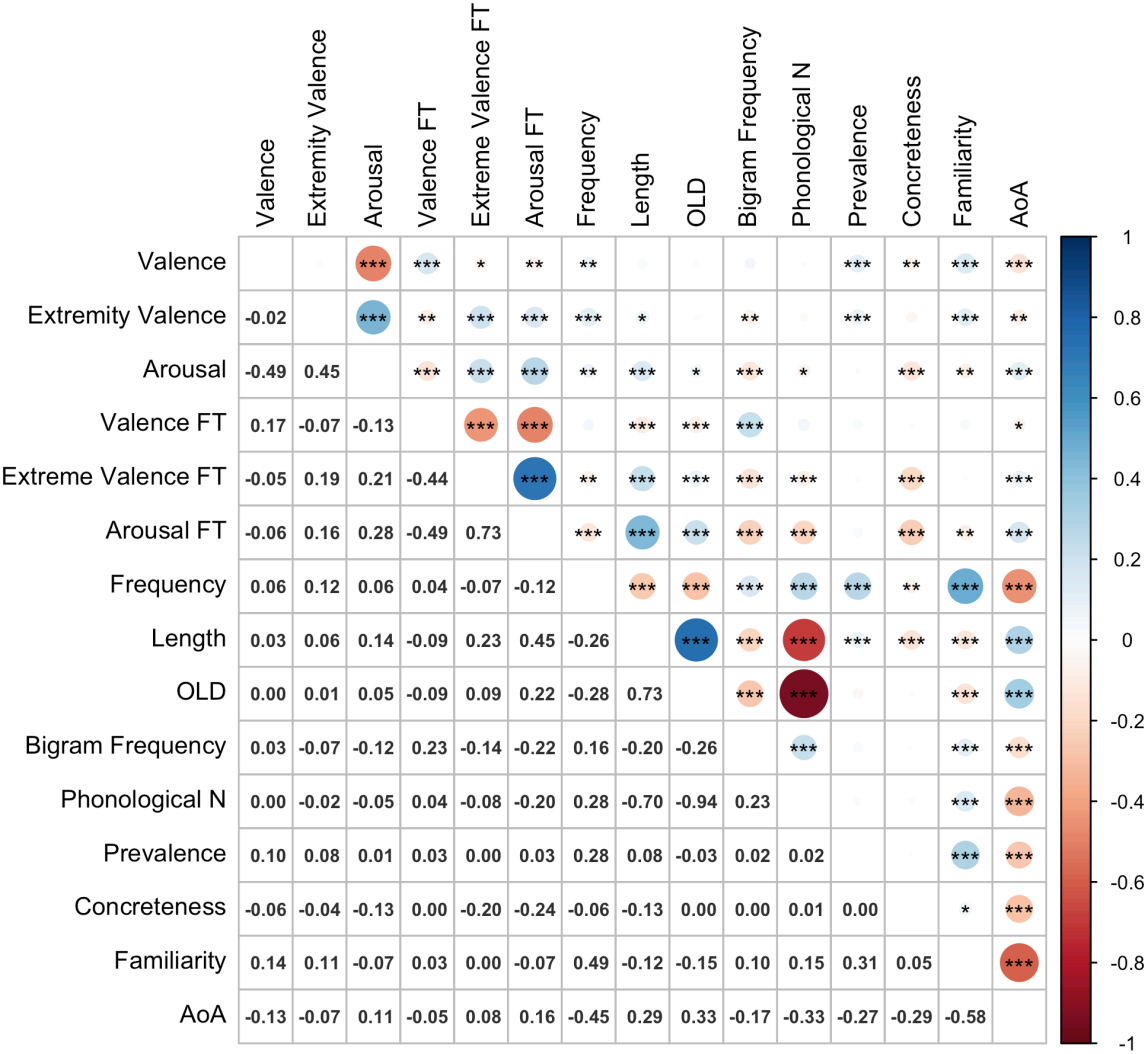
Correlations among variables (*n* = 1862). FT: Form Typicality; AoA: Age of Acquisition; OLD: orthographic Levenshtein distance; Phonological N: Number of Phonological Neighbours.

The zero-order correlations among the continuous variables in the regression analyses are shown in Figure 5. Valence form typicality was positively correlated with valence (*r* = .17, *p* <.001), and negatively correlated with both emotionality (*r* = –.07, *p* < .001), and arousal (*r* = –.13, *p* < .001). More typical forms for valence tended to comprise fewer letters, were closer to their orthographic neighbours in terms of Levenshtein distance and acquired earlier in life. Emotionality form typicality was positively correlated with emotionality (*r* = .19, *p* <.001) and arousal (*r* = .21, *p* <.001) and negatively correlated with valence (*r* = –.05, *p* < .05). More typical forms were less frequent and comprised less frequent bigrams, were further from their orthographic neighbours in terms of Levenshtein distance and had fewer phonological neighbours. They were also more abstract in meaning, comprised more letters and were acquired later in life. Finally, arousal form typicality was positively correlated with arousal (*r* = .28, *p* <.001) and emotionality (*r* = .16, *p* <.001), and negatively correlated with valence (*r* = –.06, *p* <.01). More typical word forms showed similar relationships with the other variables to the emotionality typicality values, but also referenced less familiar meanings. Of note, the form typicality values for arousal and emotionality were strongly positively correlated (*r* = .73, *p* <.001), while both were moderately negatively correlated with the form typicality values for valence (*r* = –.49, *p* <.001, and *r* = –.44, *p* <.001, respectively).

The regression results are presented in Tables 6, 7 and 8. The control predictor variables explained 2.85% of variance in the valence ratings, with emotionality and arousal ratings together explaining an additional 18.4%. The form typicality variables were also able to explain a further 1.3% of variance, although form typicality for emotionality did not contribute significantly. Form typicality for valence was the strongest predictor, followed by form typicality for arousal. The control predictor variables explained slightly more variance in the emotionality ratings (5.4%), with valence and arousal ratings together significantly contributing an additional 32% of variance. Only form typicality for emotionality and arousal contributed significantly toward another 1.3% of variance, with the former being the stronger predictor of the two. The pattern of findings for the arousal ratings was similar. Here the control predictor variables explained 7.8% of variance, with the valence and emotionality ratings contributing an additional 49.2%. The form typicality variables significantly contributed an additional 0.5% of variance, however, form typicality for emotionality was not a significant predictor. Form typicality for arousal was a stronger predictor than form typicality for valence.

**Table 6:** Regression coefficients from analysis of valence (*n* = 1862).


MODEL COMPARISON	*ESTIMATE*	*STD. ERROR*	*t*	*ADJUSTED R^2^*	*ΔR^2^*

*Step 1 (control predictors)*				0.0285***	

Intercept^†^	6.375	0.513	12.434***		

Length	0.055	0.027	2.048*		

OLD	–0.105	0.096	–1.097		

Phonological Neighbours	0.001	0.003	0.179		

Mean bigram frequency	0.000	0.000	0.673		

Zipf frequency	–0.156	0.051	–3.075**		

Prevalence	0.058	0.108	0.540		

Concreteness	–0.163	0.034	–4.838***		

Familiarity	0.076	0.035	2.157*		

Age of Acquisition	–0.103	0.023	–4.415***		

*Lexical Category:*					

Adjective	–0.270	0.120	–2.258*		

Adverb	–0.209	0.239	–0.877		

Verb	–0.292	0.096	–3.029**		

*Step 2*				0.469***	0.184***

Emotionality	–0.179	0.039	–4.646***		

Arousal	–0.792	0.028	–28.742***		

*Step 3*				0.482***	0.013***

TypValence	0.211	0.030	6.946***		

TypEmotionality	0.035	0.032	1.100		

TypArousal	0.111	0.041	2.690**		


^†^Represents reference level. TypValence: form typicality for valence; TypEmotionality: form typicality for Emotionality; TypArousal: form typicality for arousal. * p < .05; ** p < .01; *** p < .001.

**Table 7:** Regression coefficients from analysis of emotionality (*n* = 1862).


MODEL COMPARISON	*ESTIMATE*	*STD. ERROR*	*t*	*ADJUSTED R^2^*	*ΔR^2^*

*Step 1 (control predictors)*				0.054***	

Intercept^†^	–0.300	0.347	–0.866		

Length	–0.004	0.017	–0.219		

OLD	0.060	0.063	0.946		

Phonological Neighbours	–0.005	0.002	–2.459*		

Mean bigram frequency	0.000	0.000	–3.291**		

Zipf frequency	0.201	0.033	6.029***		

Prevalence	0.077	0.072	1.074		

Concreteness	0.043	0.023	1.862		

Familiarity	0.009	0.023	0.376		

Age of Acquisition	0.011	0.015	0.744		

*Lexical Category:*					

Adjective	0.260	0.075	3.484***		

Adverb	–0.363	0.165	–2.198*		

Verb	0.332	0.061	5.443***		

*Step 2*				0.374***	0.320***

Valence	–0.093	0.022	–4.230***		

Arousal	0.413	0.035	11.959***		

*Step 3*				0.386***	0.012***

TypValence	0.011	0.021	0.522		

TypEmotionality	0.141	0.024	5.837***		

TypArousal	–0.070	0.029	–2.429*		


^†^Represents reference level. TypValence: form typicality for valence; TypEmotionality: form typicality for Emotionality; TypArousal: form typicality for arousal. * p < .05; ** p < .01; *** p < .001.

**Table 8:** Regression coefficients from analysis of arousal (*n* = 1862).


MODEL COMPARISON	*ESTIMATE*	*STD. ERROR*	*t*	*ADJUSTED R^2^*	*ΔR^2^*

*Step 1 (control predictors)*				0.078***	

Intercept^†^	3.651	0.376	9.713***		

Length	0.025	0.020	1.243		

OLD	0.058	0.067	0.872		

Phonological Neighbours	–0.002	0.002	–0.614		

Mean bigram frequency	0.000	0.000	–3.602***		

Zipf frequency	0.286	0.039	7.415***		

Prevalence	–0.034	0.083	0.406		

Concreteness	0.049	0.026	1.894		

Familiarity	0.051	0.027	–1.893		

Age of Acquisition	0.075	0.018	4.227***		

*Lexical Category:*					

Adjective	0.333	0.087	3.821***		

Adverb	–0.099	0.211	–0.467		

Verb	0.540	0.067	8.098***		

*Step 2*				0.570***	0.492***

Valence	–0.388	0.023	–16.509***		

Emotionality	0.388	0.034	11.546***		

*Step 3*				0.575***	0.005***

TypValence	0.056	0.020	2.761**		

TypEmotionality	–0.025	0.023	–1.071		

TypArousal	0.141	0.028	5.075***		


^†^Represents reference level. TypValence: form typicality for valence; TypEmotionality: form typicality for Emotionality; TypArousal: form typicality for arousal. * p < .05; ** p < .01; *** p < .001.

These results confirm that the form typicality values from Study 1 each contribute significant unique variance to predicting the affective content of Spanish words.

## Study 3: Written lexical decision

As we noted in the Introduction, only two mega-studies have investigated the influence of affective variables on Spanish word processing using the lexical decision task (LDT). Both Rodríguez-Ferreiro and Davies (2019) and Haro et al. (2024) reported a graded effect of valence on LDT RTs, with positive words responded to more quickly than neutral and negative words. Rodríguez-Ferreiro and Davies (2019) did not observe an effect of arousal, while Haro et al. (2024) did, as well as an interaction between arousal and valence indicating that arousal delayed the identification of positive words whereas it speeded the recognition of negative words. However, neither study controlled for rating disagreement (see Pollock, 2018) or investigated emotionality. Here, we investigated whether valence, emotionality and arousal and their corresponding measures of form typicality were significant predictors of LDT latencies and accuracy using Haro et al.’s (2024) megastudy dataset. We expected to replicate the prior findings of a graded effect of valence on RTs and Haro et al.’s findings for arousal (as we used the same dataset). In addition, we hypothesized that the corresponding measures of affective form typicality would be significant predictors of RTs.

## Methods

### Participants

Haro et al.’s (2024) online megastudy was conducted with 918 participants (641 female; mean age = 27.51 years, range = 17 – 70, SD = 11.05).

### Materials

The materials comprised the same set of 1862 words from Study 2 and their corresponding lexico-semantic variables. LDT RTs and mean error rates for these words were sourced from Haro et al.’s (2024) megastudy.

### Design and analysis

We adopted a similar hierarchical linear regression approach to de Zubicaray et al. (2023; Experiment 3), performing separate regressions with robust standard errors (Wilcox, 2016) with two dependent variables from Haro et al. (2024): mean RTs and mean error rates, using the packages *estimatr* (Blair et al., 2022) and *lmtest* (Zeileis & Hothorn, 2002). In each analysis, we entered the control predictor variables in Step 1. Next, we entered valence, emotionality and arousal in Step 2 followed by their interactions in Step 3. In Steps 4 and 5, we entered the corresponding measures of form typicality followed by their interactions (Model “a”). All predictor variables were mean-centred. We then repeated these steps (Model “b”), reversing their order of entry (i.e., form typicality followed by valence measures). Note that adopting this approach allows valence, emotionality and arousal and their corresponding measures of form typicality to each explain both their unique and shared variance with the others.

## Results and Discussion

**Figure 6:**
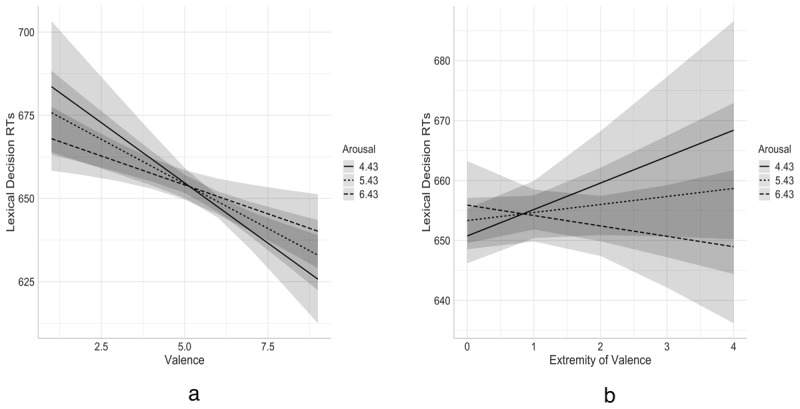
Added variable plot showing the relationships between lexical decision RTs and (a) valence and (b) Emotionality as a function of arousal after controlling for the lexico-semantic predictor variables. Shaded area shows 95% confidence intervals.

The results for the LDT RTs and mean error rates are presented in Tables 9 and 10. Together, the control predictor variables accounted for 52.1% of variance in RTs. When entered first, the affective ratings contributed a small amount of additional variance (0.1%). However, only valence contributed significantly to the model, with more positive words being responded to more quickly, replicating prior results of mega-studies conducted in Spanish (Haro et al., 2024; Rodríguez-Ferreiro & Davies, 2019) and English (Kuperman et al., 2014). In addition, interactions between arousal and both valence and emotionality contributed significant additional variance (0.2%), replicating Haro et al (2024; cf., Rodríguez-Ferreiro & Davies, 2019). Figure 6 shows these interactions after controlling for the lexico-semantic predictor variables entered in Step 1.

**Table 9:** Regression coefficients from item-level analyses of LDT RTs (*n* = 1862).


MODEL COMPARISON	*ESTIMATE*	*STD. ERROR*	*t*	*ADJUSTED R^2^*	*ΔR^2^*

*Step 1 (Control variables)*				0.521***	

*Step 2a (Valence variables)*				0.522***	0.001*

Valence	–2.523	1.084	–2.327*		

Emotionality	–2.338	1.486	1.573		

Arousal	0.031	1.527	0.021		

*Step 3a (Interactions)*				0.524***	0.002**

Valence × Arousal	1.879	0.876	2.146*		

Emotionality × Arousal	–3.073	1.170	–2.627**		

*Step 4a (Form Typicality variables)*				0.525***	0.001

TypValence	–0.051	1.563	–0.032		

TypEmotionality	–3.050	1.592	–1.916+		

TypArousal	3.779	1.983	1.906+		

*Step 5a (Interactions)*				0.525***	0.000

TypValence × Arousal	0.607	1.351	0.449		

TypEmotionality × Arousal	–1.264	1.376	–0.919		

*Step 2b (Form Typicality variables)*				0.521***	0.000

TypValence	–0.706	1.554	–0.454		

TypEmotionality	–3.240	1.559	–2.078*		

TypArousal	3.627	1.988	1.824+		

*Step 3b (Interactions)*				0.522***	0.001

TypValence × Arousal	0.555	1.354	0.410		

TypEmotionality × Arousal	–1.394	1.382	–1.009		

*Step 4b (Valence variables)*				0.523***	0.002

Valence	–2.544	1.107	–2.298*		

Emotionality	–1.967	1.535	1.281		

Arousal	–0.309	1.531	0.202		

*Step 5b (Interactions)*				0.525***	–0.005**

Valence × Arousal	1.926	0.876	2.199*		

Emotionality × Arousal	–2.958	1.175	–2.517*		


TypValence = Form Typicality for Valence; TypEmotionality = Form Typicality for Emotionality; TypArousal = Form Typicality for Arousal. + p < .07; * p < .05; ** p < .01; *** p < .001.

**Table 10:** Regression coefficients from item-level analyses of LDT error rates (*n* = 1862).


MODEL COMPARISON	*ESTIMATE*	*STD. ERROR*	*t*	*ADJUSTED R^2^*	*ΔR^2^*

*Step 1 (Control variables)*				0.454***	

*Step 2a (Valence variables)*				0.454***	0.000

Valence	–0.031	0.133	–0.234		

Emotionality	0.078	0.194	0.402		

Arousal	–0.328	0.197	–1.663		

*Step 3a (Interactions)*				0.454***	0.000

Valence × Arousal	0.175	0.121	1.451		

Emotionality × Arousal	–0.049	0.173	0.280		

*Step 4a (Form Typicality variables)*				0.453***	–0.001

TypValence	0.025	0.249	0.099		

TypEmotionality	–0.028	0.249	–0.114		

TypArousal	–0.21	0.311	–0.066		

*Step 5a (Interactions)*				0.453***	0.000

TypValence × Arousal	0.329	0.192	1.713		

TypEmotionality × Arousal	0.271	0.211	1.283		

*Step 2b (Form Typicality variables)*				0.453***	–0.001

TypValence	0.029	0.247	0.116		

TypEmotionality	–0.025	0.244	–0.103		

TypArousal	–0.073	0.308	–0.237		

*Step 3b (Interactions)*				0.453***	0.000

TypValence × Arousal	0.320	0.191	1.673		

TypEmotionality × Arousal	0.265	0.210	1.263		

*Step 4b (Valence variables)*				0.453***	0.000

Valence	–0.042	0.136	–0.308		

Emotionality	0.095	0.201	0.470		

Arousal	–0.340	0.200	–1.697		

*Step 5b (Interactions)*				0.453***	0.000

Valence × Arousal	0.174	0.121	1.442		

Emotionality × Arousal	–0.057	0.174	–0.327		


TypValence = Form Typicality for Valence; TypEmotionality = Form Typicality for Emotionality; TypArousal = Form Typicality for Arousal. * p < .05; ** p < .01; *** p < .001.

When entered next, the form typicality variables together were not significant, with typicality for emotionality and arousal likewise not reaching significance in the model. None of the form typicality interactions contributed significant proportions of variance. A similar pattern of findings emerged when the form typicality variables were entered first, although typicality for emotionality was now a significant predictor and typicality for arousal again approached but did not reach significance (p = .068). When entered after the form typicality variables, valence remained a significant predictor. Interestingly, when entered last, both the interactions with arousal now significantly *reduced* the amount of variance explained (–0.5%). In the analyses of mean error rates, the lexico-semantic control predictor variables explained a significant proportion of the variance (52.1%). However, none of the affective ratings, their corresponding form typicality values, or their interactions explained any significant additional variance.

## General Discussion

Previous studies have provided limited evidence for affective sound symbolism across languages. The present study investigated whether more extensive systematic mappings might exist between the phonological features of Spanish words and their affective meanings, i.e., affective form typicality. We found clear evidence of affective form typicality using a large sample of Spanish words with reasonable rating agreement for valence, emotionality and arousal measures. These affective form-meaning mappings occurred at the level of both phonemes and phonetic features and remained significant when controlling for a range of lexico-semantic variables.

Research on affective sound symbolism has focussed mainly on relationships between valence and specific phonemes, with only a few studies conducted in Spanish. We replicated reports of initial and final phonemes significantly predicting valence in Spanish, as well as negative words being over-represented in terms of fricatives and nasals in their initial phonemes (Adelman et al., 2018; Calvillo-Torres et al., 2024). Overall, form variables were able to predict approximately 3% of the variance in valence ratings of unaffixed words. Here, more positive words also tended to comprise more phonemes overall, including more alveolar, lateral, mid and unrounded sounds. They also had more bilabial and velar sounds in their initial phonemes and final phonemes that were also more likely to be stressed and comprise more lateral and unrounded sounds. In this line, the unrounded vowel /i/ has been repeatedly associated with positive feelings, which possibly arises from an overlapping in the muscles used to smile and to articulate this phoneme (Rummer & Schweppe, 2019; Sidhu et al., 2022; but see Wagenmakers et al., 2016). Also, bilabial sounds like /l/ or /m/ are used more often in texts expressing pleasantness and are associated with feelings of tenderness and sweetness (Fónagy, 1991; Whissell, 1999). Conversely, negative words tended to be shorter. In addition to comprising more fricatives and more nasals in their first phoneme, they also had more alveolar final sounds. The acoustic properties of nasalized and fricative sounds (e.g., changes in the spectral balance associated with the articulatory effort or the noisy airflow) have been shown to elicit unpleasant feelings (Kienast & Sendlmeier, 2000; Louwerse & Qu, 2017).

Overall, the current results bear some similarity to those recently reported for English in which form features predicted approximately 2% of the variance in valence ratings (de Zubicaray et al., 2023). More positive words in English likewise tend to have more bilabial and velar sounds in their initial phoneme and negative words comprise more fricatives and nasal sounds in their initial phoneme. However, the languages differ with respect to how the final phoneme is stressed for valence; positive and negative words being more likely to have final stress in Spanish and English, respectively. In Table 11 we summarise the form features predictive of valence that were common or unique to these two languages.

**Table 11:** Form features predictive of valence common and unique to English and Spanish.


FORM FEATURE	*ENGLISH*	*SPANISH*

*Both languages*		

Number fricative	–	–

Final phoneme	+	+

First Phoneme bilabial	+	+

First Phoneme velar	+	+

First Phoneme nasal	–	–

Final Stress Position	–	+

*Spanish*		

Length		–

Number phonemes		+

Number labiodental		–

Number alveolar		+

Number affricate		+

Number lateral		+

Number mid		+

Number unrounded		+

Initial Phoneme		+

First Phoneme labiovelar		+

Final Phoneme alveolar		–

Final Phoneme fricative		–

Final Phoneme lateral		+

Final Phoneme unrounded		+

*English*		

Number stop	–	

First Phoneme stop	–	

Final Phoneme labiodental	+	

Number syllables	+	

Initial Stress Position	–	

Medial Stress Position	–	


*Note*: + valence; – negative valence.

To our knowledge, our study is the first to demonstrate form-meaning mappings for emotionality (emotional intensity regardless of polarity) in Spanish (e.g., Adelman & Estes, 2013), explaining approximately 4% of variance. More emotionally intense words tended to be longer, and were associated with more nasal, fricative, approximant and voiceless sounds overall, despite having fewer voiceless sounds in their initial phoneme. They also had more final phonemes comprising dental and alveolar sounds. Conversely, more neutral words tended to have more syllables, as well as more bilabial, dental, alveolar, palatal, labiovelar, and velar sounds. Their initial phonemes also comprised more alveolar sounds, while their final phonemes had more nasal, fricative, lateral and trill sounds. Again, form-meaning mappings were more extensive in Spanish than English, and accounted for more variance (4% versus 1.3%, respectively; de Zubicaray et al., 2023). This difference might reflect the more consistent associations between phonemes and their sound in Spanish compared to English. Only the numbers of bilabial sounds and syllables were common to emotionality in both languages: Stronger emotional intensity was associated with fewer bilabials in both languages but fewer versus more syllables in Spanish and English, respectively. Of note, more emotionally intense words in English tend to be associated with more voiced sounds in their initial phoneme (de Zubicaray et al., 2023), indicating differential involvement of the vocal chords across the two languages for these meanings.

In English, Warriner et al.’s (2013) arousal ratings show excessive inter-individual variability such that calculation of valid form-meaning mappings is not possible at the lexicon-level (de Zubicaray et al., 2023). Interestingly, this was not the case for Stadthagen-Gonzalez et al.’s (2018) norms, which showed that arousal is represented in terms of a nomothetic (i.e., population level; see Kuppens et al., 2013) category of affective meaning in the Spanish lexicon. In addition, form-meaning mappings were able to explain relatively more variance (~6%) in arousal than in valence or emotionality ratings. We were able to replicate Calvillo-Torres et al.’s (2024) finding that highly arousing words in Spanish tend to comprise more fricatives. Although we could not replicate their findings concerning approximants and arousal qualities, approximants did show a relationship with emotionality in the present study, as we noted above. Of note, Calvillo-Torres et al. did not control for rater disagreement or redundant affixes. This may at least partially explain these differences across studies and emphasises the need to consider these variables when investigating sound-symbolic associations. Here, more arousing meanings tended to have longer forms comprising more labiodental and fricative sounds. Their initial phonemes were also over-represented in terms of dental, affricate and unrounded sounds. The finding that fricatives like /s/ or/f/ tend to occur more often in high-arousing words is consistent with prior reports in German (Schmidtke & Conrad, 2018; Ullrich et al., 2016). The articulation of fricatives produces a hissing sound that may elicit feelings of alertness and excitement given its resemblance with threatening sounds uttered by some animals (e.g., snake’s hiss; Conrad et al., 2022). Words with less arousing/more calming connotations had more velar, lateral, mid and unrounded sounds, and more voiceless sounds in their initial phoneme. In addition, their final phonemes were more likely to be stressed and comprised more lateral, open, and mid sounds.

We also investigated whether affective form typicality varied according to Part of Speech (grammatical category). We found that adjectives were the most typical forms for positive valence, which is also the case for English (de Zubicaray et al., 2023). This can be considered consistent with the use of adjectives to directly reference immediate feelings and emotional states (e.g., Béligon, 2020; Galati et al., 2008; Pérez-Sánchez et al., 2021). In addition, these forms tended to be acquired earlier in life. In English, adjectives were also the most typical forms for strong emotionality, whereas in Spanish verbs were the most typical forms. Verbs were also the most typical forms for high arousal. A reason for this difference across languages is not immediately apparent. However, various researchers have noted that Spanish differs to English by having language features that promote and expand affective connotations (e.g., Llabre, 2021). For example, the morphological markers to create diminutives and augmentatives in Spanish seem to play a role in conveying emotions (Hinojosa et al., 2022). Also, the subjunctive mood is used more frequently in Spanish than English to add affective information to the infinitive form of verbs. Interestingly, more typical forms connoting emotionality and arousal were also acquired later in life.

Overall, the results highlight key differences between the constructs of affective sound symbolism and affective form typicality. The former construct is primarily concerned with a small set of phonemes that convey perceptuomotor analogies with affective content, such as sharing of the muscles used to smile (Rummer & Schweppe, 2019; Sidhu et al., 2022), and so are often represented across languages. Conversely, cues to statistical relationships between form features and affective connotations are based on phonological regularities within a given language, so are more likely to be language-specific and more extensive (Dingemanse et al., 2015). As Spanish is a more phonotactically constrained language than English, this is likely to explain the different and more extensive relationships we observed with phonological features.

Systematic relationships between form and meaning have been shown to aid learning of linguistic categories (such as emotional valence) during language acquisition (Dingemanse et al., 2015; Haslett & Cai, 2023; Monaghan et al., 2014). Whereas more typical forms for positive valence were acquired earlier in life, we observed the opposite relationship for emotionality and arousal. It is well known that vocabulary size increases with age (Keuleers et al., 2015). Emotion regulation is also a core skill that advances as we age, peaking during adolescence and continuing to mature into adulthood (Gross, 2015; Livingstone & Isaacowitz, 2021). This might explain why word forms that comprise cues associated with heightened arousal and emotionality are acquired later. However, it should be acknowledged that the correlations between age of acquisition and all three affective variables and their corresponding form typicality measures were quite weak.

A fundamental question concerns the pressures that motivated these systematic sound-affective meaning regularities, which remain elusive. Some authors have speculated that systematic associations between forms and emotional meanings reflect evolutionary skills to integrate multi-modal inputs with affective experiences (Imai & Kita, 2014; Vinson et al., 2021). There is evidence indicating that animals generate harsh and rough vocalizations in aggressive encounters with other animals, whereas harmonic and pure tone-like sounds are associated with friendly and approaching behaviours (Di Stefano & Spence, 2022; Sidhu & Pexman, 2018). Similar associations between roughness and distress situations have been observed in the acoustic structure of baby cries (Koutseff et al., 2018), or in scream vocalizations signalling alarm in both children and adults (Arnal et al., 2015). Of note, recent evidence indicates that rough sounds involve synchronous activity between superior temporal brain regions underlying sound perception and limbic areas critically involved in the appraisal of danger (Arnal et al., 2019). Non-arbitrary relationships between word forms and affective meanings might therefore have evolved to provide cues to increase the speed and accuracy of communicating messages that signal events with a potential relevance for survival (Adelman et al., 2018). However, sound-affective meaning associations are also clearly shaped by the constraints imposed by the acoustic and phonological profiles of different languages.

While the present study has provided evidence for affective form typicality being a statistical property of the Spanish lexicon, it is worth noting that the overall proportion of variance explained by the form variables was relatively small. With lexico-semantic variables entered first in the regression models in Study 2, the amount of significant variance explained further reduced to 1.3% in valence and emotionality and to 0.5% for arousal. This reinforces the view that the primary channels for communicating emotional content are facial expressions and suprasegmental features in speech (affective prosody; e.g., Neves et al., 2021), which offer more flexibility. Prosodic features also vary considerably across languages, with Spanish having a more complex intonation stress pattern structure than English (e.g., Alcoba & Murillo, 1998).

The findings from the lexical decision task in Study 3 for valence were consistent with those of previous studies despite our use of a smaller sample of words with good rating agreement. More positive words were associated with faster responses (e.g., Haro et al., 2024; Rodríguez-Ferreiro & Davies, 2019; Siakaluk et al., 2016), a finding that possibly arises from a preference for using positive words and the higher elaboration or semantic richness of information in memory for positive words compared to both negative and neutral words (Dodds et al., 2015; Kuperman et al., 2014). We were also able to replicate Haro et al.’s finding of an interaction between arousal and valence (see also Citron et al., 2014; Larsen et al., 2008 for converging evidence; cf., Rodríguez-Ferreiro & Davies, 2019), showing that high arousal facilitated the recognition of negative words and conversely delayed the recognition of positive words. We also found a similar interaction with emotionality. These findings align with the avoidance-approach hypothesis, which argues that high arousal and negative valence elicit withdrawal strategies whereas low arousal and positive valence elicit approach responses (Robinson et al., 2004). The congruency in avoidance behavioural tendencies in negative high arousal would lead to facilitated processing. In contrast, impaired responses would be expected when individuals have to face incongruent action tendencies when identifying positive high-arousing words. Our findings suggest that this conflict is more evident for words with more intense affective referents. However, the overall contribution of affective variables to predicting performance was relatively weak, explaining only a fraction of a percent of the variance, comparable to the findings with English words (de Zubicaray et al., 2023). Affective form typicality was also a weak and inconsistent predictor of performance, with only form typicality for emotionality emerging as a significant predictor of performance, with more typical forms responded to more quickly. In English, affective form typicality was shown to explain relatively more variance in auditory LDT and recognition memory performance, which might also be the case for Spanish (de Zubicaray et al., 2023). Unfortunately, megastudy data is not currently available for these tasks in Spanish.

## Conclusions

A number of studies have provided evidence of affective sound symbolism across various languages. We explored whether the affective connotations of Spanish words might be associated with more extensive statistical relationships with phonological/phonetic features. Our findings demonstrate that affective form typicality is a statistical property of the Spanish lexicon, complementing similar findings in English. The need to examine non-arbitrary associations between form and meaning across languages has been emphasized (Elsen et al. 2021) in line with prior claims about the generalizability and universality of sound-symbolic effects (Dingemanse et al., 2015). Here we partially replicated prior reports of statistical regularities found in English and German, such as the over-representation of fricatives in words conveying negative emotions or high-arousing concepts. In contrast, we found differences in how the final phoneme is stressed for valence and more extensive associations between form and affective meaning in Spanish than English. A promising avenue for future research might entail investigating systematic mappings between forms and affective connotations for basic or discrete emotions (e.g., happiness, anger, disgust) for which norms in Spanish are now available for 9000 words (e.g., Calvillo-Torres et al., 2024; Hinojosa et al., 2023).

## Data Accessibility Statement

All data and analysis scripts are publicly available at: https://osf.io/mxhnq/.

## Footnotes

The term *systematicity* has also been used to refer to form variations resulting from morphological inflections and derivations (e.g., negating affixes such as *in-* in *infeliz*; see Haslett & Cai, 2023). We use the term *typicality* to connote statistical regularities in surface form features associated with a category distinction in unaffixed Spanish words (affective content).

We also conducted identical analyses using the full set of words including the redundant affixed forms, the results of which are available in the *Supplementary Materials* in our OSF archive.

We included length in our analyses as it was the only orthographic variable contributing to the form typicality regression models.
